# Financial implications of a hospital early mobility program

**DOI:** 10.1186/2197-425X-3-S1-A758

**Published:** 2015-10-01

**Authors:** K Bognar, JW Chou, D McCoy, AL Sexton Ward, J Hester, P Guin, AB Jena

**Affiliations:** Precision Health Economics, Los Angeles, United States; Hill-Rom, Chicago, United States; University of Florida, Gainesville, United States; Harvard Medical School, Boston, United States

## Introduction

Decades of advancements in critical care medicine have led to dramatic mortality and morbidity reductions in intensive care units (ICU). Unfortunately, significant advances in neuromuscular and neurocognitive functional recovery have lagged behind [1], despite broad evidence demonstrating that patients who survive critical care admissions may have long-term function declines, and increased long-term morbidity and mortality [2].

Early mobility programs (EMP) have been shown to improve patient clinical outcomes; however, a lack of evidence associated with financial outcomes has served as a barrier to investment for program implementation [3].

## Objective

To evaluate the financial benefits/challenges associated with the published clinical outcomes of EMPs in the US ICU environment.

## Methods

Using literature based clinical outcome estimates of EMPs for ICU patients, we developed a financial impact model and simulated the impact of introducing an EMP in an ICU on costs to hospitals, third-party payers, and capitated healthcare delivery systems. The model includes the following key variables: annual number of ICU admissions by ventilation status and insurance type; improvements in clinical and administrative outcomes e.g. length of stay (LOS), days on ventilator, and rate of hospital readmission; cost savings associated with the clinical and administrative outcomes; and start-up and operating costs.

## Results

The total net present value over a seven-year time horizon of an EMP for a US hospital with 1000 yearly ICU admissions exceeds $2.3 m. The yearly cost-of-care savings generated by reducing ICU and Non-ICU for both ventilated and non-ventilated patients and the number of days on ventilation for a hospital is approximately $927,000. The impact of EMPs on hospital readmissions generates an additional $93,000 annual savings by reducing US hospital readmission penalties. The model also suggests that EMPs generate even greater value for third-party payers and capitated health systems.

## Conclusions

EMPs may generate substantial clinical improvements in ICU patients and reduce costs to hospitals, payers, and capitated health care delivery systems. Even when the expected clinical effectiveness of an EMP is reduced by 20% the estimated net present value was positive by the second year of the program.

## Grant Acknowledgment

This study was supported by Hill-Rom Corporation.
